# The oral microbiome, pancreatic cancer and human diversity in the age of precision medicine

**DOI:** 10.1186/s40168-022-01262-7

**Published:** 2022-06-15

**Authors:** Kelly M. Herremans, Andrea N. Riner, Miles E. Cameron, Kelley L. McKinley, Eric W. Triplett, Steven J. Hughes, Jose G. Trevino

**Affiliations:** 1 Department of Surgery, University of Florida College of Medicine, P.O. Box 100286, Gainesville, FL 32610 USA; 2 Department of Microbiology and Cell Science, University of Florida, P.O. Box 110700, Gainesville, FL 32611-0700 USA; 3 Division of Surgical Oncology, Virginia Commonwealth University, 1200 E Broad St, Richmond, VA 23298-0645 USA

**Keywords:** Disparities, Microbiota, Cancer, Oral health, Periodontitis, Genetics, Race, Socioeconomics, Smoking

## Abstract

Pancreatic cancer is a deadly disease with limited diagnostic and treatment options. Not all populations are affected equally, as disparities exist in pancreatic cancer prevalence, treatment and outcomes. Recently, next-generation sequencing has facilitated a more comprehensive analysis of the human oral microbiome creating opportunity for its application in precision medicine. Oral microbial shifts occur in patients with pancreatic cancer, which may be appreciated years prior to their diagnosis. In addition, pathogenic bacteria common in the oral cavity have been found within pancreatic tumors. Despite these findings, much remains unknown about how or why the oral microbiome differs in patients with pancreatic cancer. As individuals develop, their oral microbiome reflects both their genotype and environmental influences. Genetics, race/ethnicity, smoking, socioeconomics and age affect the composition of the oral microbiota, which may ultimately play a role in pancreatic carcinogenesis. Multiple mechanisms have been proposed to explain the oral dysbiosis found in patients with pancreatic cancer though they have yet to be confirmed. With a better understanding of the interplay between the oral microbiome and pancreatic cancer, improved diagnostic and therapeutic approaches may be implemented to reduce healthcare disparities.

Video Abstract

**Supplementary information:**

Supplementary information accompanies this paper at 10.1186/s40168-022-01262-7.

## Introduction

Despite significant advancements in cancer therapeutics, pancreatic cancer (PC) remains one of the deadliest malignancies with an estimated overall 5-year survival rate of 11% [1]. Over the next year alone, 47,050 people are projected to die from PC in the US [2]. It is anticipated that by 2030 rates will double, making it the second leading cause of cancer-related mortality [2]. Globally, PC is the cause of death for an estimated 441,083 individuals and the sixth leading cause of cancer death worldwide [3]. The majority (80 to 90%) of patients diagnosed with PC are incurable at the time of presentation due to advanced disease [4]. Further, the small percentage of patients eligible for curative surgical resection often experience early recurrence and subsequent death [4].

The disease burden of PC does not affect populations uniformly as significant healthcare disparities exist in prevalence, treatment and mortality. Thus, an important opportunity in improving patient care is identification of the biologic and environmental factors that negatively contribute to PC evolution and patient outcomes. Identified factors include genetics, race/ethnicity, socioeconomic status (SES), smoking, and age [5]. Determining the mechanisms that drive these disparate outcomes will ultimately help to improve prevention strategies and targeted therapeutics.

Over the past two decades, the advent of low-cost, genetic sequencing has allowed for investigation into the unexplored world of the human oral microbiome and its contribution to systemic disease [6]. Though often overshadowed by its gastrointestinal counterpart, the oral microbiome is the second largest microbiome in the human body. It is home to over 700 different species of bacteria as well as fungi, viruses and protozoa [7]. Made up of the hard and soft palate, floor of the mouth, lips, tongue, teeth, gingiva, and buccal mucosa, the oral cavity provides a complex environment for microbial and host interactions [8]. Communicating through signaling molecules, microbiota adapt to environmental change, defend against invasion and create biofilms to aid in colonization [9].

The human oral microbiome consists of both a core and variable component [7]. A core microbiome is similar across healthy individuals [10], whereas the variable microbiome is uniquely shaped by external influences and changes in physiology [11]. The overall composition of the oral microbiome changes throughout development based on a culmination of inherited and environmental factors [12–14]. Through joint evolution with the host, microbiota adapt to play an intricate part in digestion, metabolism, detoxification, and immune regulation, all of which can contribute to the development and progression of disease [15].

Oral microbial imbalance or maladaptation, otherwise referred to as dysbiosis, has been found to influence both locoregional and systemic diseases [16]. Oral dysbiosis has been correlated locally with periodontal disease, dental caries and oral cancers [17–19] as well as a wide array of systemic diseases including diabetes, cardiovascular disease, rheumatoid arthritis, Alzheimer’s disease, osteoporosis, pulmonary disease, and pre-term delivery [20, 21]. Furthermore, recent studies have drawn attention to the pathophysiology between the human oral microbiome and cancer development and progression [22]. Oral dysbiosis has been associated with cancers of the esophagus, liver, stomach, breast, lung, colon and rectum. However, correlations between the oral microbiome and PC have arguably been the most widely studied [23].

Established in 2008, the Human Microbiome Project (HMP) and expanded Human Oral Microbiome Database (eHOMD) were created by the National Institutes of Health (NIH) in order to facilitate the characterization of the human microbiome and analyze its role in human health and disease [24, 25]. Creation of these large databases has enabled researchers to compare and investigate the beneficial and detrimental roles of the oral microbiome in human health [26]. As new associations between the oral microbiome and human health emerge, host-microbiome interactions may become integrated into the science of precision medicine. Utilizing these novel techniques, investigators have the potential to incorporate these ideals into the development of new patient-specific diagnostic and therapeutic targets. The goals of this review are threefold: 1) to report the known associations between oral health, the oral microbiome and PC, 2) to discuss how human diversity effects the composition of the oral microbiome and 3) to explore potential mechanisms behind the interplay of human diversity, the oral microbiome and PC development.

### Oral health and pancreatic cancer

The oral microbiome has recently become of interest for its role in the development and treatment of PC. The association between poor oral health and the development of PC first began as astute clinical observation, but is now supported by several studies, including meta-analyses [26, 27]. Though some studies did not account for confounding variables such as smoking, it is important to acknowledge that risk factors affect patient biology systemically and are intertwined in the development of PC [28, 29]. These initial association studies (Table 1) established the groundwork for further exploration into the pathophysiology of periodontal disease and PC development. Using this background knowledge, further studies were initiated to investigate the correlation between oral health and PC on a microscopic level.

**Table 1:** Associations between oral health and pancreatic cancer

Author (year)	Participants (n)	Study	Findings	Location
Stolzenberg-Solomon *et al*. (2003) [30]	29104 male smokers	Alpha-Tocopherol, Beta-Carotene Cancer Prevention (ATBC) Study	Tooth loss is associated with PC.	Finland
Huang *et al.* (2016) [31]	19924 participants	Swedish Cancer and Total Population registers	Oral lesions and tooth loss are associated with PC.	Sweden
Michaud *et al.* (2007) [32]	48375 US male health professionals	The Health Professionals Follow-Up Study (HPFS)	Periodontal disease is associated with PC. Tooth loss is not associated with PC.	United States
Chang *et al.* (2016) [33]	139805 individuals with periodontal disease and 75085 controls	National Health Insurance Research Database of Taiwan	Periodontitis is associated with PC in individuals over the age of 65.	Taiwan
Gerlovin *et al.* (2019) [34]	59000 Black American women	Black Women’s Health Study (BWHS)	Periodontitis and tooth loss are associated with PC.	United States

To explore the correlations between poor oral health and PC, Stolzenberg-Solomon *et al*. first performed a cohort analysis of male smokers [30]. They found an association between edentulism (tooth loss) and incidence of PC (HR = 1.63; 95% CI: 1.09- 2.46). Though this study was well-powered, its inclusion criteria of only male smokers limited generalizability [30]. Huang *et al.* further investigated the associations between poor oral health and PC development [31]. They followed individuals over 28 years after a baseline dental exam and found that those with fewer teeth and oral lesions had up to an 80% excess risk of PC, while adjusting for confounding variables [31]. Supporting evidence of a correlation between periodontal disease and PC was also reported by Chang *et al.* [33] Investigators evaluated the PC risk of individuals with periodontal disease within the National Health Insurance Research Database (NHIRD) of Taiwan. They found a positive association between periodontitis and PC in individuals over the age of 65 (HR= 1.55; 95% CI: 1.02–2.33) but this correlation was not established in those younger than 65 years of age (HR= 0.83; 95% CI: 0.52–1.34) [33]. Further, Gerlovin *et al.* utilized the Black Women’s Health Study (BWHS) comprised of initial oral health questionnaires from Black American women who were followed over an average of 10 years [34]. They found that periodontitis and tooth loss, disproportionately common in Black Americans, was associated with an increased risk of PC [34]. In contrast to the above studies, Michaud *et al*. did not find any association between tooth loss and PC [32]. They analyzed both the impact of edentulism and periodontal disease on PC through the prospective analysis of US male health professionals. Their results however did yield a significant association between periodontal disease and the development of PC (RR = 1.64, 95% CI = 1.19- 2.26; *p* = 0.002), when strictly adjusting for cigarette smoking and additional, potentially confounding variables [32].

### The oral microbiome and pancreatic cancer

In the last two decades, the launch of next generation, high throughput DNA sequencing has allowed for a more thorough view of the oral cavity through evaluation of the oral microbiota [8]. The bacteria, fungi and viruses that were not previously recognized through standard culturing technique were able to be rapidly identified and analyzed. As a result, a new world of microbial discovery began as investigators explored the oral microbiome and its association to PC (Table 2) [35].

**Table 2:** Changes in the oral microbiome associated with pancreatic cancer

Author (year)	Participants (n)	Study Type	Findings	Location
Fan *et al.* (2018) [36]	361 PC and 371 matched controls	Prospective cohort study	*P.gingivalis* and *A. actinomycetemcomitans* are associated with PC. Phylum Fusobacteria and genus *Leptotrichia* are associated with decreased PC risk.	United States
Michaud *et al.* (2013) [37]	405 PC and 416 matched controls	Prospective cohort study	Antibodies to *P. gingivalis* increased PC risk twofold, specifically to strain ATCC 53978.	European Countries
Vogtmann *et al.* (2020) [38]	273 PC and 285 matched controls	Comparative Study	*Enterobacteriaceae*, *Lachnospiraceae G7*, *Bacteroidaceae*, or *Staphylococcaceae* were associated with PC. *Haemophilus* decreased odds of PC.	Iran
Farrell *et al.* (2012) [39]	10 PC and 10 healthy controls Validation: 28 PC, 27 chronic pancreatitis, 28 controls	Comparative study	*N. elongata* and *S. mitis* are decreased and *G. adiacens *is increased in PC. *G. adiacens* and *S. mitis* are increased in PC when compared to chronic pancreatitis.	United States
Lin *et al.* (2013) [40]	12 PC, 3 pancreatitis, 12 healthy controls	Comparative study	*Bacterioides* genus are increased in PC. *Corynebacterium* and *Aggregatibacter* are decreased in PC.	United States
Torres *et al*. (2015) [41]	108 PC, 78 “other diseases,” 22 healthy controls	Comparative Study	Increased ratio of *Leptotrichia* to *Porphyromonas ﻿*in PC. No difference in *S. mitis* and *G. adacians* in PC.	United States
Wei *et al.* (2020) [42]	41 PC, 69 healthy controls	Comparative Study	*Streptococcus* and *Leptotrichina* increased in PC. *Veillonella* and *Neisseria* increased in controls.	China
Olson *et al*. (2017) [43]	34 PC, 39 IPMN, 58 controls	Comparative Study	Increased proportion of Firmicutes phylum in PC.	United States

First, in a landmark study utilizing the Cancer Prevention Study II (CPSII) and Prostate, Lung, Colorectal and Ovarian (PLCO) prospective databases, Fan *et al.* analyzed the oral microbiome of patients that eventually went on to develop PC versus matched controls [36]. Given the prospective nature of these databases, oral wash samples were collected up to 10 years prior to cancer diagnoses and matched to controls based on age, sex, race and calendar year of collection. They found that individuals who harbored the bacterial pathogens *Porphyromonas gingivalis* and *Aggregatibacter actinomycetemcomitans* within their oral microbiome had an increased risk for developing PC (OR =1.60, 95% CI 1.15-2.22; OR=2.20, 95% CI 1.16-4.18, respectively). They also determined that patients with the bacterial phylum Fusobacteria and genus *Leptotrichia* had a decreased risk of PC (OR=0.94, 95% CI 0.89–0.99; OR=0.87 95% CI 0.79–0.95, respectively) [36]. *P. gingivalis* and *A. actinomycetemcomitans* are known pathobionts, naturally benign organisms that become pathologic under certain conditions. Thus, the association of periodontitis and PC development was further strengthened. More importantly, these pathobionts could serve as potential biomarkers for the identification of patients at higher risk for PC.

Second and furthering support for *P. gingivalis* as a potential bacterial signature for risk for PC, Michaud *et al.* evaluated pre-diagnostic blood samples from patients that subsequently developed PC compared to matched healthy controls within the European Prospective Investigation into Cancer and Nutrition (EPIC) Study [37]. They measured antibodies to a preselected panel of known oral bacteria and found that individuals with high levels of antibodies against *P. gingivalis* (>200ng/mL) had a higher risk of developing PC (OR= 2.14; 95% CI 1.05 - 4.36). This correlation specifically applied to the strain of *P. gingivalis* ATCC 53978, known for the pathogenicity of its capsule. Alternatively, individuals with high levels of antibodies to common commensal oral bacteria had a 45% lower risk of developing PC [37].

Third, in order to compare the oral microbiome of patients currently diagnosed with PC to healthy controls, Vogtmann *et al.* classified the oral microbiota of PC patients and their matched controls [38]. They found that *Enterobacteriaceae*, *Lachnospiraceae G7*, *Bacteroidaceae*, and *Staphylococcaceae* were increased in patients with PC whereas the presence of *Haemophilus* was increased in controls [38]. Farrell *et al.* used the Human Oral Microbe Identification Microarray (HOMIM) to similarly differentiate patients with PC from controls, including subsequent validation of findings in an independent cohort of patients with PC, chronic pancreatitis and healthy controls [39]. Using the bacterial composition of decreased *N. elongata* and *S. mitis* and increased *G. adiacens* as a biomarker, patients with PC could be differentiated from healthy controls with a 96.4% sensitivity and 82.1% specificity. Additionally, authors found a significant increase in *G. adiacens* and *S. mitis* in patients with PC when compared to patients with chronic pancreatitis [39]. The bacterial species *N. elongata* has been implicated in periodontal disease [44] and *G. adiacens* is an opportunistic pathobiont that is often found in settings of systemic inflammation [45]. Lin *et al.* explored the microbial composition of a relatively small cohort of patients with PC (*n*=13), pancreatitis (*n*=2) and healthy controls (*n*=12) in their published abstract [40]. Their data suggest that pathobionts within the *Bacterioides* genus are more abundant in patients with PC. They found that *Corynebacterium* and *Aggregatibacter* are underrepresented in PC patients [40]. These findings contradict those of Fan *et al.*, which indicate that *A. actinomycetemcomitans*, a species within the *Aggregatibacter* genus, are associated with increased risk of PC development [36]. However, they do not specify the species of *Aggregatibacter* evaluated and sample size was comparatively low [40]. To explore the oral microbial signature in PC, Torres *et al*. analyzed the composition of the oral microbiota in the saliva of patients with PC, “other diseases” and healthy controls [41]. They found a significantly higher ratio of *Leptotrichia* to *Porphyromonas *in PC patients [41]. Interestingly, *Leptotrichia* species are opportunistic pathogenic bacteria that are often found in immunocompromised patients [42, 46]. In addition, they found no difference in *S. mitis* and *G. adacians* levels, contrasting data reported by Farrell *et al.* [39]. In order to specifically study the oral dysbiosis associated with PC in Chinese subjects, Wei *et al*. evaluated the oral microbiome of patients with PC and healthy controls by clinical presentation ﻿[42]. They found that PC was associated with carriage of *Streptococcus* and *Leptotrichina*, with *Veillonella* and* Neisseria* found more commonly in healthy controls. When analyzing patients’ oral microbiome based on clinical presentation, findings of* Porphyromonas*, *Fusobacterium*, and *Alloprevotella* were associated with bloating, *Prevotella* was associated with jaundice, *Veillonella* was associated with *bilirubinuria*, *Neisseria* and *Campylobacter* were associated with diarrhea and *Alloprevotella* was associated with vomiting [42].

Finally, Olson *et al*. analyzed saliva samples of patients with PC, intraductal papillary mucinous neoplasms (IPMNs) and healthy controls [43]. IPMNs are cystic neoplasms of the pancreas that have a known potential for malignant transformation, dictated in part by their location in the pancreas. Investigators observed an increase in the Firmicutes phylum in patients with PC and an increased level of Proteobacteria phylum in controls. Notably, differences between PC and IPMN patients mirrored those between PC and healthy controls thus suggesting that the oral microbiome may be a suitable marker to differentiate patients with premalignant and malignant lesions [43].

**Fig. 1:**
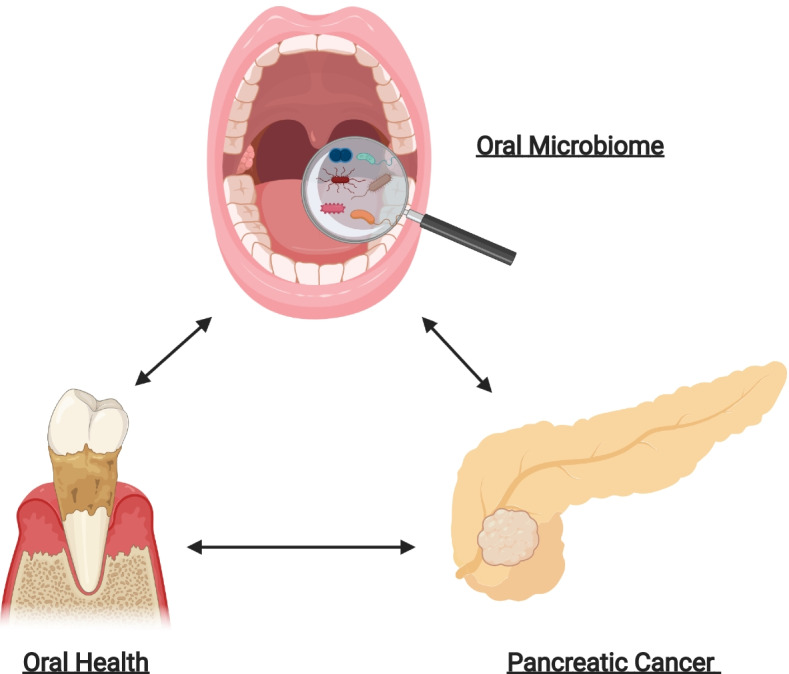
The interplay of the oral microbiome, oral health and pancreatic cancer: The oral microbiome, oral health and pancreatic cancer are intricately related, though mechanisms have yet to be elucidated

In summary, poor oral health, oral microbial dysbiosis and the development and progression of PC are interlinked (Fig. 1). However, the underlying mechanisms of the oral microbiota’s influence in PC diagnosis and treatment have yet to be elucidated. Thus, these data beg for further research, particularly as it relates to mechanisms, human diversity and the implementation of precision medicine.

### Human diversity and the oral microbiome

**Fig. 2:**
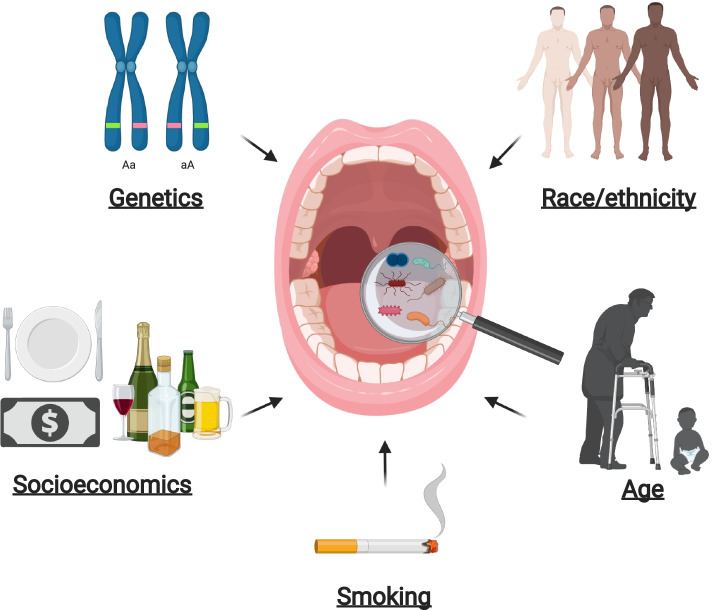
Individual heterogeneity impacts the oral microbiome: Human diversity shapes the composition of the oral microbiome. Environmental influences including genetics, race/ethnicity, socioeconomics, smoking and age affect the makeup of an individual’s oral microbiome

Throughout the process of human development, the oral microbiota becomes representative of an individual, based on both their genetic background and environment (Fig. 2). Current literature addressing the impact of human heterogeneity on the development of the oral microbiome and its subsequent influence on PC is limited; available data is summarized below.

### Genetics

A family history of PC is found in an estimated 5-10% of patients diagnosed with PC. Though several genes have been identified, most familial PC clusters exhibit no known heritable factors [47]. Similarly, the oral microbiome demonstrates heritability and is influenced by an individual’s genotype. In fact, when the first humans migrated, so did the microorganisms that made up their microbiome [48]. Through the influence of vertical transmission and environmental impact, bacterial strains have been shown to represent human ancestry better than traditional human genetic markers [49]. Genetic analysis of four strains of *S. mutans*, a bacterial species common to the oral cavity, identifies ancestral migration patterns and geographic heritage [48].

This heritability is further demonstrated by identical and fraternal twin studies exploring the influence of the host genotype on the composition of the oral microbiota in both adults and children [50–52]. Through genomic analysis, Demmitt *et al.* identified loci on chromosomes 7 and 12 that significantly impacted the phenotypic composition of the oral microbiota [50]. They also found that heritability of the oral microbiome persisted despite changes in cohabitation. Gomez *et al.* analyzed the microbiome of subgingival plaques, identifying an inheritance pattern in monozygotic and dizygotic twins [51]. In addition, they found that the composition of heritable microorganisms decreased significantly with age. Friere *et al.* reiterated the heritability of certain oral microbial species including *Actinomyces* and *Capnocytophaga* in monozygotic twins and *Kingella* in dizygotic twins [52]. However, they did emphasize that environmental changes exert greater influence on the oral microbiome than genetic predisposition [52].

Taken together, the oral microbiome reflects heritage and has the potential to be utilized in precision medicine. The oral microbiome could feasibly be incorporated into genetic risk scores in the future. However, further -omics level interrogation is needed to characterize its interplay between heritable genes, environmental cues and PC.

### Race and ethnicity

In the US, disparities in PC prevalence, treatment and mortality disproportionally affect racial and ethnic minority populations [53, 54]. Black Americans have a higher prevalence of PC, present with more advanced disease and have increased mortality rates when compared to other racial-ethnic groups [55]. Racial and ethnic disparities are multifaceted and have the potential to be influenced by cultural norms, diet, geography, bias, and genetics [56]. The culmination of these factors aid in the design of each unique oral microbiome, tailored to an individual’s background. Mason *et al.* identified ethnicity-specific microbial communities within the oral microbiome [57]. Using a machine-learning classifier, they were able to characterize an individual’s ethnicity through the analysis of their oral microbiota [57].

In order to further explore racial differences in the oral microbiome, Yang *et al.* analyzed saliva samples from African Americans (AA) and European Americans (EA) in low-income communities [58]. They found significant differences in 32 bacterial taxa, including four known periodontal pathobionts *P. gingivalis*, *Prevotella intermedia*, *Treponema denticola*, and *Filifactor alocis.* They found that AA individuals had higher richness of Bacteroidetes and lower levels of Actinobacteria and Firmicutes. They then performed genome-wide single nucleotide polymorphism (SNP) analysis to estimate ancestry, finding that all 32 bacterial taxa were correlated with the percentage of African ancestry [58]. Correlating these findings with the aforementioned studies, *P. gingivalis* was found to be increased in both patients with African ancestry and those with PC [36]. Schenkein *et al.* compared bacterial samples of the subgingival microbiota in Black and White individuals with periodontitis [59]. They found evidence that *P. gingivalis* is more prevalent in Black adults with periodontitis [59]. Providing supporting evidence of racial and ethnic differences in the oral microbiome, Sirinian *et al.* evaluated oral bacteria of school-aged children and adolescents [60]. They compared Caucasian, Hispanic and Asian-American children, finding that two or more pathogenic bacteria were detected in 20% of Hispanics, 12% of Asian-Americans and none in Caucasians [60]. Finally, Ebersole *et al.* calculated specific antigenic diversity of *P. gingivalis* between races and ethnicities [61]. They demonstrated interracial/ethnic diversity in the strains of *P. Gingivalis* between subgroups of Black, White, Hispanic and Asian individuals. White individuals had decreased levels of antibodies to almost all *P. gingivalis* strains, suggesting a lower abundance of *P. gingivalis* in the oral microbiome. These pathogenic bacteria potentially play a role in the development of PC and may help explain the racial and ethnic disparities in PC incidence and treatment [62].

Correlations between racial and ethnic disparities, systemic disease and the human microbiome have been recognized, though few studies incorporate the oral microbiome [63–68]. One study by Yang *et al.* evaluated the influence of the oral microbiota on the development of colorectal cancer in AAs and EAs [69]. They identified the oral pathogens, *Treponema denticola* and *Prevotella intermedia*, to be associated with increased risk of colorectal cancer. Furthermore, this association was stronger in AAs than in EAs [63].

Unfortunately, much research focused on racial and ethnic diversity performed in the US tends to silo individuals into self-reported continental ancestry groups (i.e. African, European, Hispanic and Asian) with few studies utilizing ancestral informative markers [70]. This strategy overlooks the importance of recognizing individual biologic heterogeneity [71]. In the pursuit of personalized microbiomics, it is critical to account for diverse patient biology while still addressing healthcare disparities in race and ethnicity. Given the pervasive racial and ethnic disparities in the care and treatment of patients with PC, it is important to recognize the influence of race and ethnicity on the oral microbiome. In order to address the disparate outcomes in PC, the oral microbiome has the potential to be used in a precision medicine approach to diagnosis, prognosis and treatment.

### Socioeconomics

Socioeconomic disparities may impact housing, behavior, diet, exercise and access to affordable healthcare [72]. Socioeconomic factors play a role in PC disparities as patients with lower SES present with more progressive disease and have lower overall 5-year survival rates [73]. Likewise, the oral microbiome responds dynamically to the various factors associated with SES. Renson *et al.* sought to characterize the oral microbiome based on SES compared to other demographics [74]. They identified differences in the oral microbiome based on family income. In fact, they found that distinctive microbial variation based on SES was more profound than oral health maintenance activities [74]. Supporting this notion, Belstrøm *et al*. identified oral bacterial profiles that reflected SES [75]. In India, Bhardwaj *et al.* screened different socioeconomic classes for the presence of *Enterococcus faecalis*, a bacterium implicated in oral infections. They discovered a higher prevalence of enterococci within the oral cavity in individuals from lower socioeconomic class, though this study was significantly confounded by poor oral hygiene and smoking status [76]. Taken together, the oral microbiome is meaningfully affected by the host environment and SES plays a role in the composition of the oral microbiota. To further delineate the effect of the oral microbiome on PDC, disparities in socioeconomic backgrounds must be considered. Further research is needed to evaluate how SES is implicated in oral dysbiosis and potentially PC development.

### Smoking

Smoking is the leading modifiable risk factor in the development of PC [77]. Smokers have twice the risk of developing PC and worse overall survival. In fact, 20% of PC cases occur in smokers and they are 40% more likely to die from the disease. Following smoking cessation, the risk of pancreatic carcinogenesis returns to baseline after approximately 20 years [78].

The oral microbiome is significantly altered by all tobacco use but is best documented in the setting of cigarette smoking [79]. Smoking cigarettes affects the composition of the oral microbiota through a number of different mechanisms, both directly and indirectly. Bacteria exist within cigarettes and have the potential to modify the oral microbiota through direct inoculation [80]. In a study analyzing four different cigarette brands, Sapkota *et al.* identified 15 different classes of bacteria present within cigarettes [81]. Bacteria ranged from the microorganisms commonly found in soil to human pathogens, including *Acinetobacter*, *Bacillus, Burkholderia, Clostridium*, *Klebsiella*, and *Pseudomonas aeruginosa* [81]. It is possible that these microorganisms are inhaled through filters into the mouth and lungs to affect the local oral microbiome.

Smoking may also indirectly impact the composition of the oral microbiota through its broad immunosuppressive effects. Cigarette smoking leads to a blunted immune response on multiple levels, resulting in an overall impaired antimicrobial defense [82, 83]. This in turn may encourage the survival of pathogenic microorganisms in the oral cavity and oral dysbiosis. Smoking also changes the local environment by altering oxygen and pH levels. Through these derangements, selection occurs for microaerophilic and anaerobic microorganisms within the oral bacterial community [84].

Smoking induces dysbiosis of the oral microbiome. Culture analysis revealed significant shifts in oral bacteria and oral health in smokers [85]. As next-generation DNA sequencing evolved, investigators were able to better quantify oral dysbiosis present in smokers. Wu *et al. *evaluated oral samples of US adults in order to measure the effect of smoking on the oral microbiome [86]. They found that the oral microbiome of current smokers differed significantly from both never smokers and former smokers. After smoking cessation, the oral microbiome of former smokers reverted back to the microbiome of a never smoker with no notable differences. Through subsequent functional analysis from inferred metagenomes, they demonstrated that the microorganisms depleted in smoking were related to carbohydrate and energy metabolism as well as xenobiotic metabolism [86]. Through the salivary analysis of smokers and nonsmokers, Al-Zyoud *et al.* identified an oral bacterial composition unique to smokers [87]. In smokers, they found increased levels of the phyla Firmicutes, Proteobacteria, and Fusobacteria as well as *Streptococcus*, *Prevotella*, and *Veillonella* at the genus level [87]. Notably, levels of Firmicutes are increased in both smokers and PC patients [42].

Smoking not only affects bacterial concentrations within the oral cavity, it also impacts the formation of oral biofilms. Kumar *et al.* analyzed the marginal and subgingival plaque along with gingival crevicular fluid of smokers and nonsmokers [88]. They discovered the early colonization of oral biofilms in smokers with the pathologic bacteria *Fusobacterium, Cardiobacterium, Synergistes*, *Selenomonas*, *Haemophilus* and *Pseudomonas*. Local cytokines were elevated in the gingival crevicular fluid of smokers and a positive correlation between pathogenic bacterium within oral biofilms and a proinflammatory response was reported [88]*.*

Few studies have applied the correlation between oral dysbiosis in smokers with cancer development. In a study by Kato *et al.,* investigators sought to integrate smoking, the oral microbiome and colorectal carcinogenesis through the presence of *F. nucleatum* [89]. They identified evidence of oral microbiota distinct to smokers and individuals with colorectal cancer but were unable to link the two through *F. nucleatum* [89]. Sharma *et al*. evaluated the oral microbiota in smokers that may influence the development of head and neck squamous cell carcinoma (HNSCC) [90]. When comparing smokers with HNSCC to smokers without cancer, they established that smokers with HNSCC had higher relative abundance of *Stenotophomonas* and ﻿*Comamonadaceae* and reported higher interindividual variability with lower bacterial richness. Investigators also established that the degree of DNA damage correlated with oral dysbiosis [90].

These data present intriguing new findings that have the potential to be applied to the diagnosis, screening and treatment of PC. Though many of the early association studies controlled for smoking as a confounding variable to PC development [36–38], the oral bacteria implicated in smoking may directly or indirectly impact pancreatic carcinogenesis. Many novel opportunities exist to further explore the associations and mechanisms behind smoking-induced oral dysbiosis and PC.

### Age

Approximately 90% of PC diagnoses occur over the age of 55 [91]. The effects of aging occur in nearly all organs, including the oral cavity. The oral cavity undergoes loss of muscle tone in the hard and soft tissues, reduced salivary flow and connective tissue damage [92].

As the host undergoes the physiologic changes of aging, the microbiota of the oral cavity follow suit. Using crowdsourced data from guests at the Denver Museum of Nature & Science, Burcham *et al.* explored the differences in the oral microbiome between adults and children [93]. They found that the oral microbiome of adults had less diversity and was more affected by oral health habits than in children. Lira-Junior *et al.* compared the salivary microbiota of individuals over and under the age of 64 [94]. They found higher levels of pathogenic bacteria and increased inflammatory biomarkers in salivary samples of individuals over the age of 64 [94]. Applying these findings to multi-generational Indian families, Chaudhari *et al.* compared the microbial composition across generations [95]. Older individuals exhibited age-associated positive correlations between the genera *Treponema* and *Fusobacterium* as well as negative correlations with *Granulicatella* and *Streptococcus* [95]. Using regression models of oral microbial patterns, Huang *et al.* identified bacterial signatures that were able to predict the age range of adults within 5 years of age [96].

Changes in the oral microbiota have not only been evaluated through the quantity of life years, but also the quality. In an effort to identify early clinical manifestations of frailty in the aging, Ogawa *et al.* compared the oral microbiota of frail elderly patients living in a nursing home to healthy elderly controls [97]. Investigators found a significant difference in oral microbial composition and microbial diversity [97]. They identified a higher relative abundance of *Actinomyces*, *Streptococcus*, *Bacilli*, *Selenomonas*, *Veillonella*, and *Haemophilus* and a corresponding lower relative abundance of *Prevotella*, *Leptotrichia*, *Campylobacter*, and *Fusobacterium* in the cohort of frail nursing home residents [97]. Singh *et al.* broadly studied individuals between the ages of 70-82, dividing them into healthy and non-healthy aging cohorts [98]. Non-healthy aging individuals were defined as those with cancer, cardiovascular disease, pulmonary disease, diabetes or neurologic disease. Though many potential confounding factors exist, they found that healthy older individuals had a higher alpha-diversity, which is a measure of local bacterial species diversity, than non-healthy individuals [98].

In summary, the majority of diseases occur with increased incidence as individuals age, including periodontitis, atherosclerosis, dementia and cancer. A correlation with oral dysbiosis has been established in each of these disease states but future studies are needed to explore the intricacies of causation [99]. PC is predominantly a disease of the elderly and investigation into the role of the oral microbiome is greatly needed. Furthermore, a precision medicine approach to improving the care of PC in the aging should include the analysis of the oral microbiome.

### Mechanisms influencing human diversity, the oral microbiome, and pancreatic cancer

**Fig. 3:**
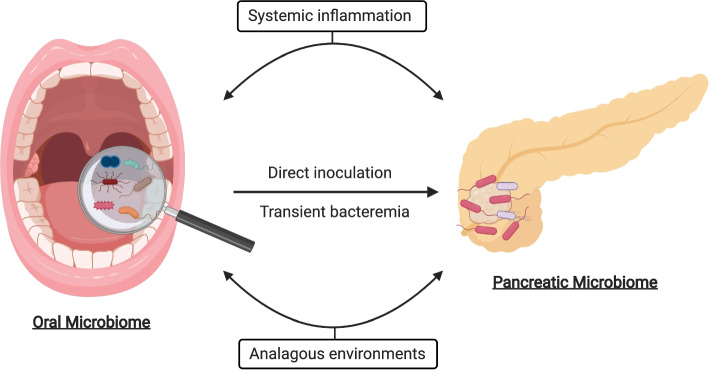
Proposed mechanisms linking the oral and pancreatic microbiomes: The composition and diversity of the oral microbiome may influence the development and treatment of pancreatic cancer through systemic inflammation, direct inoculation, transient systemic bacteremia and/or their analogous environments

There is clearly a gap in knowledge regarding associations and causations between human diversity, the oral microbiome and PC. This exciting topic creates opportunity for new research in microbiomics and the application of precision medicine. Human diversity impacts the composition of the oral microbiome which subsequently impacts overall health. The oral microbiome has the potential to play a critical role in the diagnosis and management of PC, but many questions remain unanswered. Specifically, what links the bacteria in the oral cavity to the seemingly unrelated pancreas (Fig. 3)? Do bacteria exert a direct effect or is oral dysbiosis a secondary result of systemic changes? Is there a relationship between the oral microbiome and a pancreatic microbiome?

### Analogous environments

Physiologically the oral environment, particularly the salivary gland, exhibits many similarities to that of the pancreas. They each play a role in endocrine and exocrine functions and are organized into acini and ducts. Also, both organs develop in parallel through epithelial-mesenchymal interactions and secrete bicarbonate rich digestive fluid into the alimentary canal. Through these similarities, it is possible that the oral microbiome may serve as a reflection of pancreatic changes during carcinogenesis [100].

A hallmark of PC is the presence of a dense surrounding stroma that comprises 80% of the tumor volume [101]. Our group and others have demonstrated the activation of pancreatic stellate cells into tumor-associated stroma [102]. As a result, these activated stromal components produce soluble mediators that lead to the evasion of immunosurveillance, cancer proliferation and chemoresistance [103]. Given this impact on the PC tumor microenvironment, it is conceivable that these mechanisms influence or are influenced by microbial diversity, though further research is necessary in this field [104].

### Bacterial colonization

The average human swallows approximately 1500-2000 times per day. As this occurs, saliva and oral contents pass through the esophagus, stomach and then duodenum, which is connected to the pancreas through the ampulla of Vater [105]. One possibility for colonization is that through this mechanism oral bacteria travel through the alimentary tract, reflux back into the pancreatic duct and directly seed the pancreas. Del Castillo *et al.* found that bacterial profiles of duodenal tissue were similar to that of pancreatic tissue, supporting the theory of gastrointestinal tract migration through the pancreatic duct [106]. Moreover, investigators identified the presence of bacteria characteristically found in the oral cavity within pancreatic tissue [106]. To further study correlations between the oral and pancreatic microbiota, Chung *et al.* evaluated microbial samples from the oral cavity (tongue, buccal supragingival and saliva), small intestine (duodenum and jejunum) as well as the pancreas [107]. They found statistically significant similarities between buccal, supragingival and saliva samples as well as between pancreatic duct and pancreatic tissue. Though site-specific overlap was exhibited and oral bacteria was found within pancreatic tissue, their study was limited by sample size [107]. Using germ-free mice, a human oral microbiota-associated (HOMA) model was created by transplanting human saliva into a mouse. Transplanted oral microbiota was found through source tracking to colonize the small intestines. Moreover, when HOMA mice were co-housed with fecal-transplanted mice, the bacteria found in the small intestines more closely resembled oral flora, though they did not specifically study the pancreatic microbiome [108].

Another potential mechanism of pancreatic bacterial colonization is through translocation from the gut microbiome. Given the venous and lymphatic drainage of the gut through portal system, bacteria have the potential to colonize the pancreas [35]. Preclinical animal models have shown substantial evidence that the modification of the gut microbiome through fecal microbiota transplantation affects PC tumor growth and tumor immune infiltration. Additionally, 20% of pancreatic tumor microbiota was similar to that of the gut microbiome whereas no correlation was found between nonmalignant pancreatic tissue and microbiome [109]. Further studies are needed however to correlate these findings in human disease.

Alternatively, a third potential mechanism of oral microbial transmission to pancreatic tissue is through the seeding of systemic bacteremia. Transient bacteremia occurs following tooth brushing and flossing [110]. This method of bacterial dispersion is supported by documented evidence of oral bacteria within distal sites such as atherosclerotic plaques, the brain and placenta [110].

Though the origin of the pancreatic microbiome is debated, Swidsinski *et al.* first identified the existence of bacterial biofilms in calcific pancreatic ducts [111]. The existence of the pancreatic microbiome has been explored further in recent years. Mitsuhashi *et al.* specifically sought to detect the presence of *Fusobacterium,* an oral bacterium, within pancreatic tumors [112]. In the 8.8% of patients with *Fusobacterium* colonization of their PC samples, outcomes were found to be worse [112]. Riquelme *et al.* analyzed the pancreatic microbiome of patients grouped into long-term survivors and short-term survivors of PC [109]. They found higher alpha diversity in the pancreatic microbiome in long-term survivors as well as a tumor microbiome signature (*Pseudoxanthomonas/Streptomyces/Saccharopolyspora/Bacillus clausii*) that was predictive of survival in a multi-variate analysis [109]. Gaiser *et al*. further evaluated the intracystic pancreatic microbiome using aspirated cystic fluid in surgically resected samples [113]. They found that cyst fluid from IPMNs with high-grade dysplasia was enriched with oral bacterial taxa including *F. nucleatum* and *G. adiacens.* Additionally, an elevation in intracystic bacterial DNA correlated with evidence of high-grade dysplasia and PC diagnosis [113]. Interestingly *G. adiacens* was also found to be significantly increased in the oral microbiome in patients with PC [37]. Data on *F. nucleatum*’s involvement in PC is less straightforward as decreased levels were associated with PC risk [36] and increased levels in smoking [89]. Geller *et al*. recently identified intratumoral bacteria in 76% of PC samples [114]. They found that the presence of *Gammaproteobacteria* increased resistance to gemcitabine through the expression of a long isoform of the bacterial enzyme cytidine deaminase, which converts gemcitabine into its inactive form [114]. The presence of the pancreatic microbiome both in a preclinical models and human tissue was further analyzed. Importantly, Thomas *et al.* reported the presence of pancreatic bacteria in Kras^G12D^/PTEN^lox/+^ mice as well as in benign and malignant human pancreatic surgical samples [115].

Further studies are needed to explore how the pancreatic microbiome is established and whether any direct or indirect mechanisms exist between the oral microbiome, pancreatic microbiome and pancreatic carcinogenesis. In order to understand these interactions, studies utilizing source tracking [116] in patients with PC could be potentially be performed by culturing bacteria from the oral cavity and pancreatic tumor tissue.

### Inflammation

As a key mediator of PC, inflammation has been documented as both a cause and consequence of carcinogenesis. Though the complexities of pancreatic carcinogenesis and inflammation are beyond the scope of this review, inflammation is intricately intertwined in human diversity, the oral microbiome and PC [117]. The pathogenic bacteria thriving in oral dysbiosis may modify the inflammatory milieu through direct mechanisms. *P. gingivalis*, a key suspect in oral dysbiosis and subsequent PC development, has been directly implicated in evasion of the innate and adaptive immune system. *P. gingivalis* exhibits a number of potential virulence factors and disrupts signaling pathways in order to escape immune elimination and directly inflict tissue destruction [118]. Moreover, this bacterium encourages suppression of apoptosis, tumorigenesis and cell evasion [119].

Pushalkar *et al.* demonstrated that the pancreatic microbiome also promotes oncogenesis through innate and adaptive immunosuppression [120]. Using a mouse model, pancreatic bacterial ablation was performed and downstream effects on the immune system were observed. Bacterial ablation significantly impacted the pancreatic tumor inflammasome through a reduction in myeloid-derived suppressor cells (MDSCs), increase in M1 macrophage differentiation, Th1 differentiation of CD4+ and CD8+ T-cells as well as PD-1 upregulation [120].

Despite these findings, the possibility exists that oral microbial dysbiosis and PC are two independent disease states occurring in parallel, linked by systemic inflammation. Though unknown if it is a cause or effect, periodontitis is associated with various chronic inflammatory diseases including diabetes, cardiovascular disease, obesity, and metabolic syndrome [121]. Subsequently, chronic inflammation in chronic pancreatitis is a well-documented risk factor in the development of PC [122]. Though much remains unknown, the integration of immunology with the oral microbiome and PC creates a potential arena for original research.

## Conclusions

Significant disparities in PC diagnosis, management and treatment continue to exist in diverse populations. Many unexplored mechanisms linking human diversity in PC and the oral microbiome offer a wide array of opportunities for future intervention. PC is a particularly devastating disease due to the lack of effective screening tools [123]. Changes in the oral microbiome are identifiable in PC patients prior to the onset of disease. It is easily accessible and reflects an individual’s overall health status. Given these features, the oral microbiome conceivably offers a noninvasive screening method by identifying those at higher risk of developing PC.

As more data emerges about how the pancreatic microbiome affects the efficacy of chemotherapeutics and immunotherapies, analysis of the oral microbiome also has the potential to impact the future management and treatment of patients with PC. Oral microbial sampling could potentially be utilized to choose treatment modality and gauge response in patients undergoing surgical resection, radiation or chemotherapy.

Moreover, manipulation of oral microorganisms holds promise for future therapeutic options. The correction of oral dysbiosis through the use of antibiotics, prebiotics, probiotics and microbial transplantation may potentially disrupt protumorigenic pathways [124]. The clinical application of microbiota modification also has the potential to improve treatment efficacy and sustainability in PC. When designing and implementing novel screening and therapeutic techniques in PC, it is crucial to consider the effect of human diversity and biologic heterogeneity on the oral microbiome. As the medical and research community move closer toward the new horizon of precision medicine, the oral microbiome has the opportunity to be on the forefront of medical innovation in patients with PC.

## Data Availability

Not applicable.

## Abbreviations

AA: African Americans
BWHS: Black Women’s Health Study
CD4+: Cluster of differentiation 4
CD8+: Cluster of differentiation 8
CPSII: Cancer Prevention Study II
DNA: Deoxyribonucleic acid
EA: European Americans
eHOMD: Expanded Human Oral Microbiome Database
EPIC: European Prospective Investigation into Cancer and Nutrition
HMP: Human Microbiome Project
HNSCC: Head and neck squamous cell carcinoma
HOMA: Human oral microbiota-associated
HOMIM: Human Oral Microbe Identification Microarray
IPMNs: Intraductal papillary mucinous neoplasms
MDSCs: Myeloid-derived suppressor cells
NHIRD: National Health Insurance Research Database
NIH: National Institutes of Health
PC: Pancreatic cancer
PD-1: Programmed cell death protein 1
PLCO: Prostate, Lung, Colorectal and Ovarian
SES: Socioeconomic status
SNP: Single nucleotide polymorphism
Th1: T helper cell type 1
URMs: Underrepresented minorities
US: United States
